# Removal of a Drill-Head from the Cavity of a Tooth, by Means of a Magnet

**Published:** 1845-09

**Authors:** John Harris


*Removal of a Drill-head from the Cavity of a Tooth, by means of a Magnet.*—Messrs. Editors: Whether I have been more unfortunate than other dentists in having my drills too highly tempered, I cannot say, but, in consequence of this oversight, I have occasionally had them to break, while preparing the cavities in decayed teeth for filling, leaving the burr or head in the cavity; and, in some instances, though it appeared quite loose, and upon the slightest touch would move about, its removal was attended with considerable inconvenience and loss of time, and sometimes the loss of more of the sound part of the tooth than would otherwise be necessary or desirable.

In preparing a small cavity in the grinding surface of a bicuspid of the lower jaw, for a lady, after having it nearly ready to fill, having removed the most of the diseased parts, my drill broke, leaving the burr at the bottom of the cavity; the walls of which were sound and very dense, and so close to the burr as not to admit of the passage of any small instrument between it and them, in order to remove it, and yet so loose as to be readily moved in any direction upon its axis.

Not feeling desirous of adopting the usual course resorted to in such cases, that of removing enough of the adjacent sound tooth to pass a small instrument between it and the surrounding walls, considerable time was consumed in fruitless efforts to remove it. I accidentally recollected of having seen a small magnet the same morning at a druggist's store but a few doors from my room, which I immediately procured, and with which the burr was instantly removed.

Should the like accident happen with other dentists, I believe much labor, time, and inconvenience will be saved by adopting the above plan for its removal.

Very respectfully, &c.

JOHN HARRIS.

